# Investigation of the Effects of Agomelatine in Rats with Experimental Cerebral Ischemia/Reperfusion Model

**DOI:** 10.3390/biomedicines14071603

**Published:** 2026-07-17

**Authors:** Semiha Nur Ozkaya, Furkan Yuksel, Samet Oz, Kevser Tanbek, Suat Tekin

**Affiliations:** 1 Department of Physiology, Faculty of Medicine, Kahramanmaras Sutcu Imam University, Kahramanmaras 46000, Türkiye; semihanurozkaya@ksu.edu.tr; 2 Department of Physiology, Faculty of Veterinary Medicine, Necmettin Erbakan University, Konya 42000, Türkiye; furkan.yuksel@erbakan.edu.tr; 3 Department of Veterinary Medicine, Vocational School of Health Services, Osmaniye Korkut Ata University, Osmaniye 80000, Türkiye; sametoz@osmaniye.edu.tr; 4 Department of Physiology, Faculty of Medicine, Inonu University, Malatya 44000, Türkiye; kevser.tanbek@inonu.edu.tr

**Keywords:** agomelatine, apoptosis, autophagy, cerebral ischemia

## Abstract

**Objective:** The present study aimed to investigate the neuroprotective effects of agomelatine in a rat model of cerebral ischemia/reperfusion injury, a major pathological event underlying ischemic stroke. **Methods:** Male Sprague Dawley rats included in the study were divided into four groups (n = 10/group): sham, CI/R, CI/R + 20 mg/kg Agm and CI/R + 40 mg/kg Agm. Sixty minutes of ischemia was induced in all groups except the sham group. One hour after ischemia, hydroxyethyl cellulose was administered intraperitoneally to the CI/R group, while 20 and 40 mg/kg agomelatine was administered to CI/R + Agm groups. During the three-day reperfusion period, the rats underwent neurological deficit score (NDS), rotarod, adhesive removal, and grip strength tests. At the end of the experiment, animals were decapitated and brain tissues were collected. In the collected brain tissues, infarct area was determined by TTC staining, and levels of apoptosis (Bcl-2, Bax) and autophagy (Beclin-1, ATG5, ATG7, p62) proteins were determined by Western blot. Statistical analysis of the obtained data was performed. **Results:** Compared with the CI/R group, agomelatine-treated groups showed significantly lower NDSs and adhesive removal times, as well as higher rotarod retention times and grip strength values (*p* < 0.05). Infarct area was significantly reduced following agomelatine treatment (*p* < 0.05). Agomelatine increased Bcl-2, Beclin-1, ATG5, and ATG7 protein levels while decreasing Bax and p62 expression compared with the CI/R group (*p* < 0.05). **Conclusions:** Agomelatine attenuated neurological deficits and infarct formation following CI/R injury. These neuroprotective effects may be associated with suppression of apoptosis and enhancement of autophagy-related pathways.

## 1. Introduction

Stroke is a neurological disorder characterized by temporary or permanent impairment of the brain due to blockage of the blood vessels supplying it or bleeding outside the vessels [1]. Stroke has been reported to account for 10.7% of all deaths worldwide and is recognized as the second leading cause of mortality globally [2,3]. Data from the Global Burden of Disease 2021 study have highlighted that stroke represents not only a major public health concern but also a substantial economic burden worldwide [3]. Ischemic stroke, also referred to as cerebral ischemia (CI), accounts for 65.3% of all stroke cases and occurs as a result of occlusion of one or more cerebral blood vessels, leading to a regional or complete interruption of cerebral blood flow [4]. Oxygen-glucose deprivation caused by CI, together with reoxygenation following reperfusion (R), disrupts cellular homeostasis and energy-dependent processes, thereby contributing to neuronal injury [5]. Activation of the cellular death pathways, apoptosis and autophagy, has been identified as a prominent feature within the infarcted region following CI and R injury [6]. Furthermore, CI/R-induced dysfunction of sensory and motor neurons has been shown to result in impairments in balance and motor coordination [7], somatosensory deficits [8], and reductions in grip strength [9]. Agomelatine is known to participate in physiological processes such as the regulation of suprachiasmatic nucleus activity and circadian rhythms through its agonistic effects on the melatonergic receptors melatonin receptor type 1 (MT1) and type 2 (MT2), as well as its antagonistic action on the serotonin 5-hydroxytryptamine-2C (5-HT2C) receptor [10,11]. Agomelatine, which is clinically prescribed as an antidepressant, has been shown to increase brain-derived neurotrophic factor (BDNF) levels in hippocampal tissue [12]. Agomelatine has been shown to enhance neuronal proliferation and neurogenesis in various regions of the brain, particularly in the dentate gyrus [13]. Furthermore, several studies have demonstrated that agomelatine administration attenuates neurotoxicity induced by toxic agents such as lipopolysaccharide, doxorubicin, and cisplatin [14,15,16]. Moreover, agomelatine administration has been reported to improve locomotor activity impaired by neurotoxicity [16]. Agomelatine administration has been shown to attenuate inflammation in brain regions such as the raphe nuclei, periaqueductal gray, amygdala, and nucleus accumbens in mice subjected to an in vivo diabetic neuropathy model, while also enhancing cell proliferation in dorsal root ganglion cells exposed to an in vitro diabetic neuropathy model [17]. In addition to its central effects, agomelatine exhibits several notable peripheral actions, including anti-apoptotic and autophagy-inducing properties, as well as anti-inflammatory and antioxidant activities [18]. Furthermore, it has been reported to improve various metabolic parameters [17,18]. The ability of agomelatine to enhance neuronal proliferation and neurogenesis, reduce neuroinflammation, and improve metabolic parameters associated with the pathogenesis of CI/R suggests that it may mitigate CI/R-induced injury [19]. Therefore, the present study aimed to investigate the effects of agomelatine on CI/R-induced neurological deficits and functional impairments, including somatosensory dysfunction, balance and motor coordination deficits, and loss of grip strength, as well as to evaluate apoptosis- and autophagy-related protein expression in brain tissue. By combining functional assessments with the analysis of multiple autophagy-related proteins, the present study provides novel insights into the mechanisms underlying the neuroprotective effects of agomelatine.

## 2. Materials and Methods

### 2.1. Experimental Center and Animal Ethics

All procedures performed in this study were carried out in accordance with the protocol approved by the Local Animal Ethics Committee of the Faculty of Medicine at Inonu University, as documented in Decision No. 2022/9-2 of the meeting held on 27 June 2022, under Ethics Approval No. 15128 HAYBİS. All experimental procedures and analyses were conducted at the Experimental Animals Production and Research Center and the research laboratories of the Department of Physiology, Faculty of Medicine, Inonu University.

### 2.2. Experimental Groups and Study Design

Male *Sprague-Dawley* rats (10–12 weeks old, n = 40) were randomly allocated to four experimental groups (n = 10 per group) using a computer-generated randomization procedure. The groups consisted of sham, CI/R, CI/R + 20 mg/kg Agm, and CI/R + 40 mg/kg Agm. No blinding was performed during the experimental procedures, outcome assessment, or data analysis. The sample size was determined by an a priori power analysis using G*Power software (Minitab version 16.2.0). Based on an expected mean body weight of approximately 300 g, a standard deviation of 10 g, a 4% deviation, a significance level of α = 0.05, and a statistical power of 80% (β = 0.20), the analysis indicated that a minimum of 10 animals per group was required. Animals were housed individually under controlled environmental conditions (21 ± 2 °C, 12 h light/12 h dark cycle) and had free access to standard chow and tap water throughout the study. During the first three days of the experimental period, all rats were acclimated to the behavioral tests. On day 4, rats in the sham group underwent surgical exposure of the common carotid artery (CCA) without induction of ischemia and received 1 mL of physiological saline. In the remaining groups, CI/R injury was induced by middle cerebral artery occlusion, with ischemia maintained for 60 min followed by reperfusion. Animals in the CI/R group received 1% hydroxyethyl cellulose (HEC), which served as the vehicle for agomelatine. Rats assigned to the treatment groups received a single intraperitoneal injection of agomelatine at doses of 20 or 40 mg/kg at the onset of reperfusion. Dose selection was based on previously published studies [19]. Neurological deficit scores (NDS) and behavioral evaluations were conducted on experimental days 5, 6, and 7. After completion of the final behavioral assessments on day 7, the rats were sacrificed by decapitation, and brain samples were harvested for further biochemical and histological analyses. No predefined inclusion or exclusion criteria were established. No animals, experimental units, or data points were excluded, and all animals allocated to the study were included in the final analyses.

### 2.3. Preparation of Agomelatine and Hydroxyethyl Cellulose Solutions

A 1% hydroxyethyl cellulose (HEC) solution was obtained by dissolving 1 g of HEC in 100 mL of distilled water. Agomelatine powder (Sigma-Aldrich, Saint Louis, MO, USA; Lot No. #116M4702V) was suspended in 1 mL of the prepared HEC solution and administered intraperitoneally at doses of 20 or 40 mg/kg based on the individual body weights of the rats assigned to the treatment groups [19].

### 2.4. Induction of the Cerebral Ischemia/Reperfusion Model

**Figure 1:**
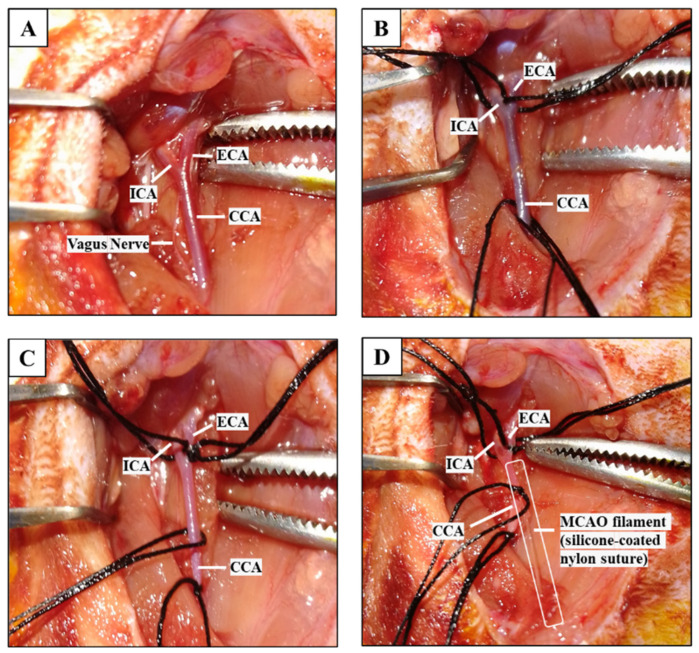
Induction of the experimental CI/R model (CCA, common carotid artery; ECA, external carotid artery; ICA, internal carotid artery; MCA, middle cerebral artery). (**A**) Isolation of the CCA, ECA, and ICA. (**B**) Placement of 5 cm-long 5–0 silk sutures beneath the CCA, ECA, and ICA. (**C**) Ligation of the CCA and ECA to interrupt blood flow. (**D**) Occlusion of the MCA by advancing a silicone-coated MCAO filament through the ICA.

Prior to surgery, anesthesia was achieved by intramuscular injection of ketamine (70 mg/kg) combined with xylazine (8 mg/kg), after which the surgical procedures were carried out [20]. The middle cerebral artery occlusion (MCAO) method was employed to generate the cerebral CI/R model. During the surgical procedure, the right common carotid artery (CCA), internal carotid artery (ICA), and external carotid artery (ECA) were identified and carefully dissected away from the vagus nerve (Figure 1A). In sham-operated animals, the procedure was terminated after vessel exposure and the incision site was closed. In the CI/R and CI/R + Agm groups, after isolation of the cervical vessels, two pieces of 5–0 silk suture (approximately 5 cm in length) were placed underneath the CCA, while a single suture was positioned beneath both the ECA and ICA (Figure 1B). Cerebral blood flow interruption was achieved by ligating the distal segment of the CCA and the proximal segment of the ECA (Figure 1C), followed by temporary clamping of the ICA with a microvascular clip. A small arteriotomy was created in the CCA about 1 mm proximal to the CCA bifurcation, and a silicone-coated monofilament (tip diameter: 0.39 mm) was inserted through the ICA branch and advanced until reaching the origin of the middle cerebral artery (MCA), thereby producing arterial occlusion (Figure 1D). Following surgery, animals were kept under close observation until complete recovery from anesthesia and were subsequently monitored for any signs of discomfort or pain. Analgesic treatment was provided in accordance with the protocols approved by the institutional animal ethics committee.

### 2.5. Neurological Deficit Scoring

**Figure 2:**
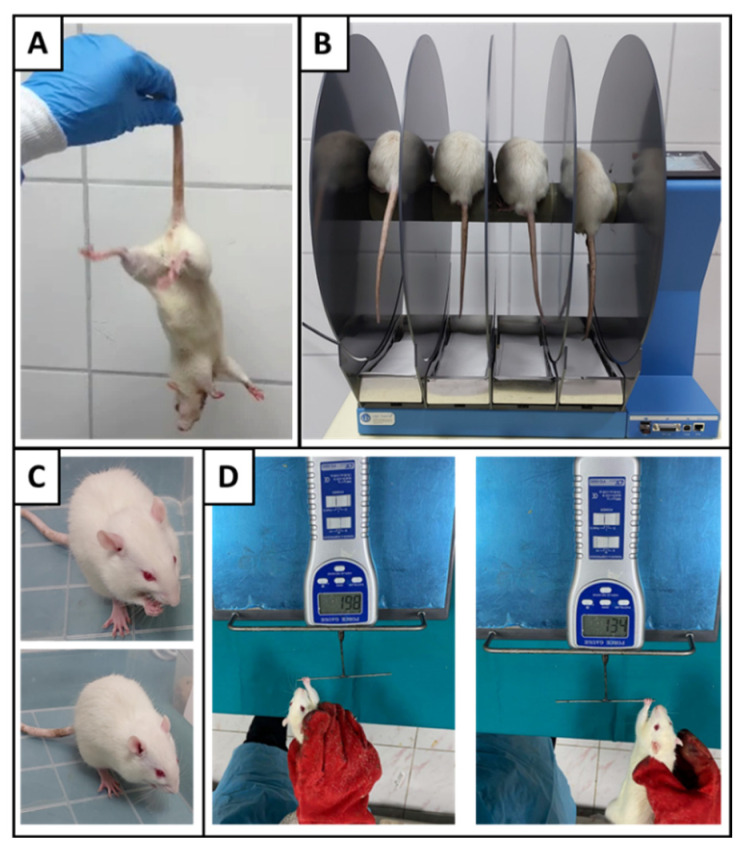
Neurological deficit scoring and behavioral assessments. (**A**) Neurological deficit scoring; (**B**) rotarod test; (**C**) adhesive removal test; (**D**) grip strength test.

Neurological deficit scoring and behavioral assessments were performed on days 5, 6, and 7 of the experiment. Measurements were performed once daily during the three-day reperfusion period, and the average values obtained over the three days were used for statistical analysis. The severity of neurological dysfunction was determined using the Bederson neurological scoring scale (Figure 2A) [21]. At 24 h after reperfusion, each animal underwent neurological evaluation, and the observed findings were assigned scores according to the classification shown in Table 1.

### 2.6. Behavioral Tests

#### 2.6.1. Rotarod Test

Motor performance and balance were assessed by recording the time each animal remained on the rotarod apparatus before falling (Figure 2B). To familiarize the rats with the procedure, a habituation phase was carried out during the first three experimental days using a rotarod device (Ugo Basile (Gemonio, Italy), Cat. No. 47750, Italy) operating at a fixed speed of 5 rpm for 15 min. Following surgery, rotarod testing was performed over three consecutive days in the accelerating mode, in which the rotational speed gradually increased from 4 to 40 rpm within 300 s. For each rat, three trials were conducted with a 5-min resting period between successive measurements [22,23].

#### 2.6.2. Adhesive Removal Test

Somatosensory performance was evaluated using the adhesive removal test, in which the time required for the animals to recognize and begin removing an adhesive stimulus was recorded (Figure 2C). Prior to surgery, rats were acclimated to the test procedure over a period of three days. Behavioral assessments were then carried out once daily for three consecutive days after the operation. For each trial, a 1 × 1 cm^2^ adhesive sticker was placed on the distal radial aspect of the left forepaw, and the interval between application and the first attempt to detect and remove the sticker was measured. A maximum observation period of 120 s was allowed for each assessment [22].

#### 2.6.3. Grip Strength Test

Forelimb muscle function was evaluated by means of the grip strength test. Animals were familiarized with the testing procedure during the first three days of the experiment (Figure 2D). Grip strength assessments were subsequently carried out on each of the three days after surgery. Using a grip strength meter (Lutron (Coopersburg, PA, USA), Cat. No. FG-5005, USA), rats were allowed to grasp the bar with each forepaw individually, and a gentle backward pull was applied until the animal released the bar. The peak force exerted at the moment of release (g) was recorded. Five measurements were obtained per day for three consecutive days, and the mean of the three highest values was used for statistical analysis. To account for inter-animal differences in body mass, grip strength values were normalized to body weight. Right and left forepaw strength-to-body weight ratios were calculated separately, and the ratio of right-to-left forepaw strength was subsequently derived by dividing the normalized value of the right forepaw by that of the left forepaw [24].

### 2.7. Animal Sacrifice and Brain Tissue Collection

Upon completion of the last behavioral evaluation, all rats were euthanized by decapitation and brain tissues were harvested. For infarct visualization, three brains randomly chosen from each experimental group were rapidly frozen on dry ice and subjected to 2,3,5-triphenyltetrazolium chloride (TTC) staining. The remaining tissue samples were preserved at −80 °C and subsequently used for Western blot analyses.

### 2.8. Tissue Analyses

#### 2.8.1. Analysis of Infarct Area by Triphenyltetrazolium Chloride Staining

**Figure 3:**
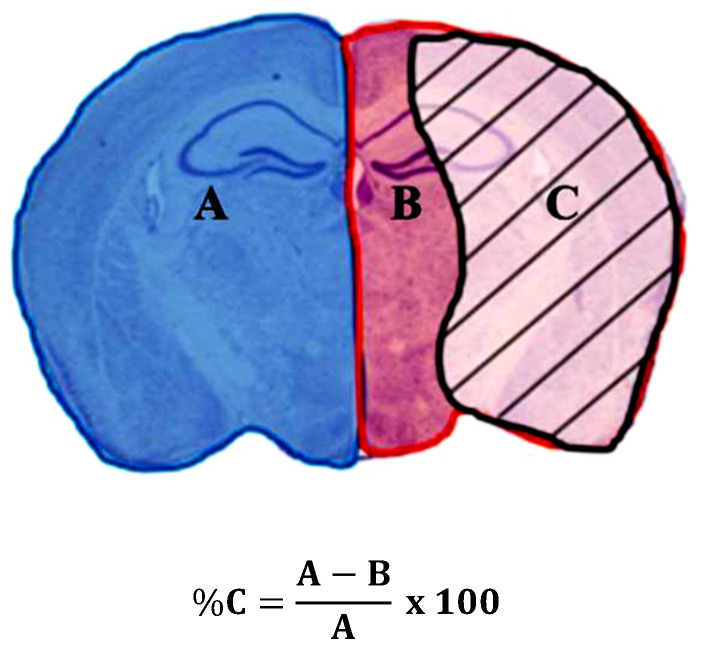
Calculation of infarct area percentage. A, total area of the contralateral hemisphere; B, non-infarcted (healthy) area of the ipsilateral hemisphere; C, infarct area.

Cerebral infarct size was evaluated using TTC staining. A 1% TTC solution was prepared by dissolving TTC powder (Serva, Heidelberg, Germany; Lot No. 211460) in phosphate-buffered saline (PBS). Brain samples were rapidly frozen on dry ice and cut into six coronal slices of 2 mm thickness spanning from +4 mm anterior to −6 mm posterior relative to the bregma. The sections were incubated in TTC solution at 37 °C for 30 min and then immersed in 4% paraformaldehyde for 12 h. After fixation, digital images of the slices were obtained and analyzed on a computer. Infarct size was calculated using ImageJ software (Version 1.54g; National Institutes of Health, Bethesda, MD, USA) according to the equation shown in Figure 3 [25].

#### 2.8.2. Analysis of Apoptotic and Autophagic Protein Levels by Western Blotting

The levels of the apoptotic proteins B-cell lymphoma 2 (Bcl-2) and Bcl-2-associated X protein (Bax), as well as the autophagy-related proteins Beclin-1, autophagy-related protein 5 (ATG5), autophagy-related protein 7 (ATG7), and p62, were determined in rat brain tissue by Western blot analysis. Brain tissues were homogenized in RIPA lysis buffer (Abcam, Cambridge, UK; Lot No. GR3289972-20) supplemented with a protease inhibitor cocktail (Abcam, UK; Lot No. GR3251345-2). Protein concentrations in the supernatants were quantified using a BCA protein assay kit (Pierce™ BCA Protein Assay Kit (Thermo Fisher Scientific, Waltham, MA, USA); Lot No. WB319447). Equal amounts of protein (50 μg) were loaded into each well of 15% sodium dodecyl sulfate-polyacrylamide gels and separated by electrophoresis. Proteins were then transferred onto membranes using a Trans-Blot Turbo RTA Transfer Kit (Bio-Rad, Hercules, CA, USA; Cat. No. 1704272). The membranes were blocked with 5% skim milk and incubated overnight at 4 °C with the respective primary antibodies. Following incubation, the membranes were washed with TBS-T and subsequently incubated with horseradish peroxidase (HRP)-conjugated secondary antibodies for 1 h at room temperature. Protein bands were visualized using an enhanced chemiluminescence substrate (Pierce™ ECL Western blotting Substrate, Thermo Fisher Scientific, Waltham, MA, USA; Lot No. YH374889) and imaged with a Fusion FX-7 imaging system (VILBER, Collégien, France). Band intensities were quantified using ImageJ software (Version 1.54g; National Institutes of Health, Bethesda, MD, USA). The expression level of each target protein was normalized to β-actin, which served as the loading control [26,27].

### 2.9. Statistical Analysis

Statistical analyses were performed using IBM SPSS Statistics for Windows, Version 22.0 (IBM Corp., Armonk, NY, USA). The Shapiro–Wilk test was used to assess the normality of data distribution. For cross-sectional between-group endpoints (e.g., apoptosis markers, autophagy markers, and behavioral test outcomes), comparisons were evaluated using the non-parametric Kruskal–Wallis H test, and pairwise comparisons were performed using the Mann–Whitney U test with Bonferroni correction. In strict accordance with data transparency standards, graphical representations were generated using GraphPad Prism software (Version 8.0; GraphPad Software, San Diego, CA, USA) as box plots with individual data points overlaid to explicitly reveal the full range and distribution of the data. No data points were excluded from the statistical analyses. A *p*-value of <0.05 was considered statistically significant. A letter-based statistical classification system was used.

## 3. Results

### 3.1. Effects of Agomelatine on Neurological Deficit Scores and Behavioral Tests

**Figure 4:**
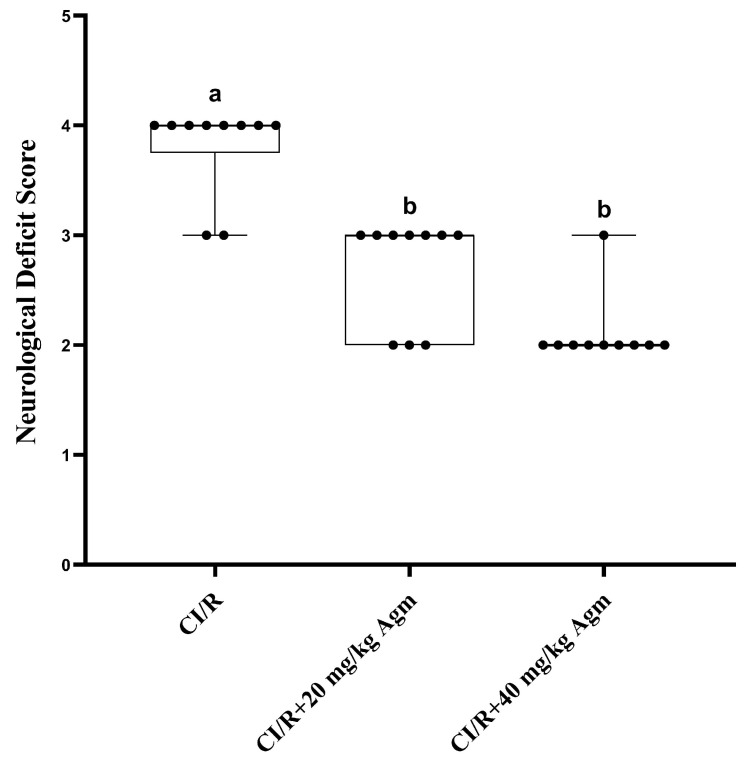
Effect of agomelatine on neurological deficit score (NDS). Data were analyzed using the Kruskal–Wallis H test followed by Bonferroni-corrected Mann–Whitney U tests for pairwise comparisons. Data are presented as box-and-whisker plots with individual data points overlaid. The box represents the interquartile range (IQR), the horizontal line indicates the median, and the whiskers represent the minimum and maximum values. Different letters indicate statistically significant differences between groups (*p* < 0.05), whereas groups sharing the same letter are not significantly different.

The effects of agomelatine on neurological deficit scores (NDS) in the CI/R model are presented in Figure 4. Statistical analysis revealed that NDS values were significantly lower in the agomelatine-treated groups compared with the CI/R group (*p* < 0.05).

**Figure 5:**
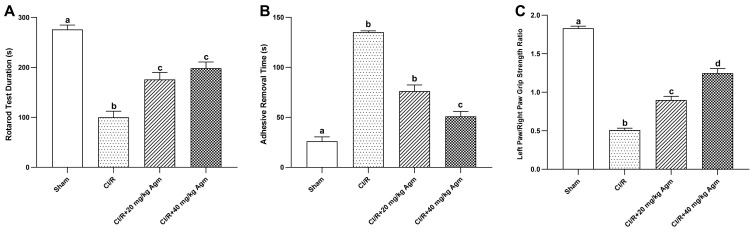
Effects of agomelatine on behavioral performance in rats subjected to CI/R injury. (**A**) Rotarod test duration, (**B**) adhesive removal time, and (**C**) left paw/right paw grip strength ratio. (Data were analyzed using the Kruskal–Wallis H test followed by Bonferroni-corrected Mann–Whitney U tests for pairwise comparisons. Data are presented as box-and-whisker plots with individual data points overlaid. The box represents the IQR, the horizontal line indicates the median, and the whiskers represent the minimum and maximum values. Different letters indicate statistically significant differences between groups (*p* < 0.05), whereas groups sharing the same letter are not significantly different.

The effects of agomelatine on behavioral performance in rats subjected to the CI/R model are summarized in Figure 5. Statistical analysis showed that CI/R significantly impaired motor coordination, somatosensory function, and grip strength compared with the sham group (*p* < 0.05). Agomelatine treatment significantly improved rotarod performance relative to the CI/R group (*p* < 0.05). Likewise, adhesive removal time was reduced and the right-to-left forepaw grip strength ratio was increased following agomelatine administration (*p* < 0.05). Notably, the 40 mg/kg dose exerted greater beneficial effects on adhesive removal performance and grip strength recovery than the 20 mg/kg dose (*p* < 0.05).

### 3.2. Effect of Agomelatine on Infarct Area

**Figure 6:**
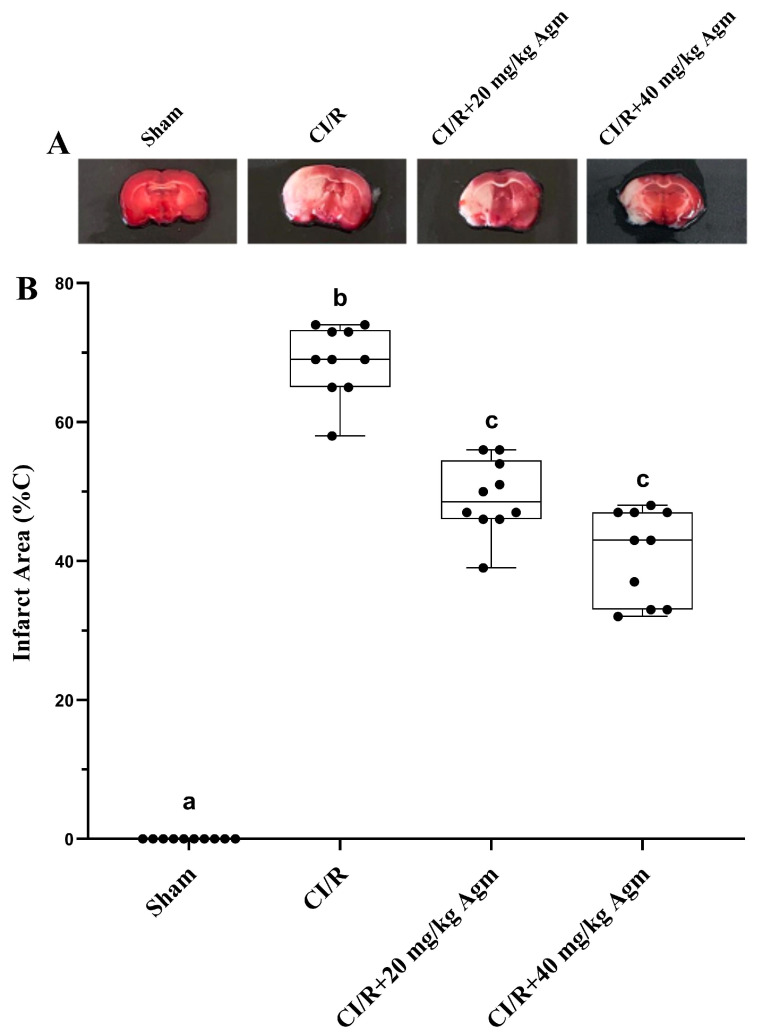
Effect of agomelatine on infarct area (%C). (**A**) Representative TTC-stained brain sections showing infarct areas. (**B**) Effect of agomelatine treatment on infarct area percentage. TTC analysis was performed using brain tissues from three animals per group (n = 3). Data were analyzed using the Kruskal–Wallis H test followed by Bonferroni-corrected Mann–Whitney U tests for pairwise comparisons. Data are presented as box-and-whisker plots with individual data points overlaid. The box represents the IQR, the horizontal line indicates the median, and the whiskers represent the minimum and maximum values. Different letters indicate statistically significant differences between groups (*p* < 0.05), whereas groups sharing the same letter are not significantly different.

Representative TTC-stained brain sections and quantitative analysis of infarct area are presented in Figure 6A and Figure 6B, respectively. TTC analysis was performed using brain tissues obtained from three randomly selected animals in each group (n = 3). No infarct area was observed in the sham group, as CI/R was not induced in these animals. Statistical analysis revealed that the percentage of infarct area was significantly lower in the agomelatine-treated groups compared with the CI/R group (*p* < 0.05).

### 3.3. Effects of Agomelatine on Apoptotic and Anti-Apoptotic Protein Levels

**Figure 7:**
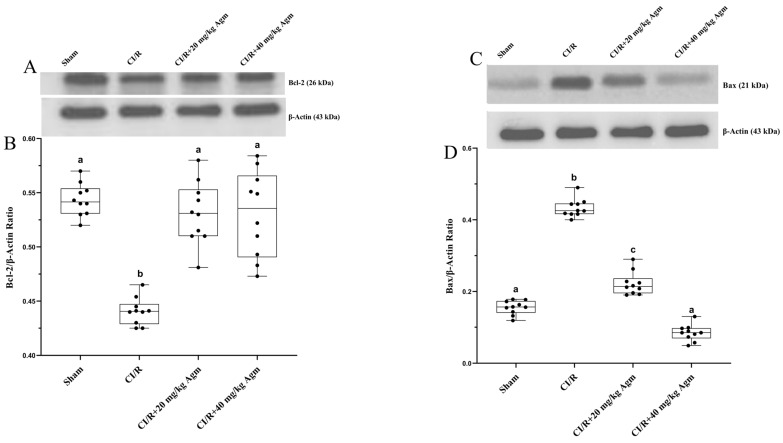
Effects of agomelatine on apoptotic protein expression in brain tissue. (**A**) Representative Western blot images of Bcl-2 and β-actin. (**B**) Densitometric analysis of Bcl-2 protein expression. (**C**) Representative Western blot images of Bax and β-actin. (**D**) Densitometric analysis of Bax protein expression. Data were analyzed using the Kruskal–Wallis H test followed by Bonferroni-corrected Mann–Whitney U tests for pairwise comparisons. Data are presented as box-and-whisker plots with individual data points overlaid. The box represents the IQR, the horizontal line indicates the median, and the whiskers represent the minimum and maximum values. Different letters indicate statistically significant differences between groups (*p* < 0.05), whereas groups sharing the same letter are not significantly different.

The effects of agomelatine on apoptotic protein expression in brain tissue are presented in Figure 7. Statistical analysis demonstrated that CI/R injury significantly decreased Bcl-2 protein levels and increased Bax protein levels compared with the sham group (*p* < 0.05). Agomelatine treatment reversed these changes, resulting in significantly higher Bcl-2 levels and lower Bax levels relative to the CI/R group (*p* < 0.05). In addition, Bcl-2 protein expression in agomelatine-treated animals was comparable to that observed in the sham group (*p* > 0.05). Notably, the reduction in Bax protein expression was more pronounced in the 40 mg/kg agomelatine group than in the 20 mg/kg group (*p* < 0.05).

### 3.4. Effects of Agomelatine on Autophagy-Related Protein Levels

**Figure 8:**
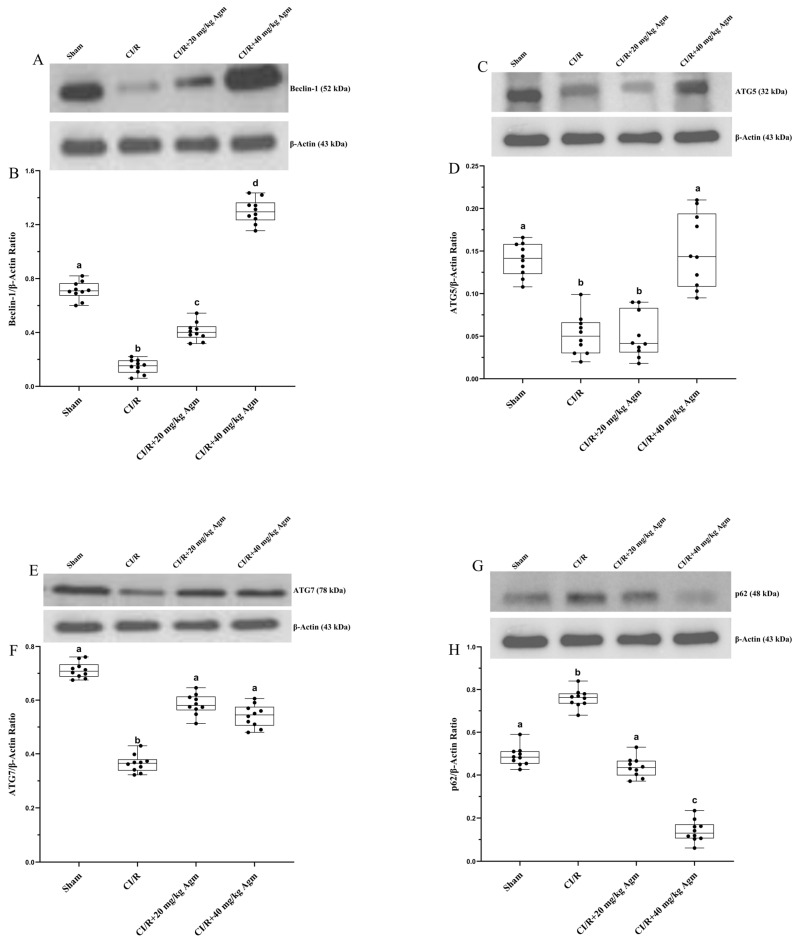
Effects of agomelatine on autophagy-related protein expression in brain tissue. (**A**) Representative Western blot images of Beclin-1 and β-actin. (**B**) Densitometric analysis of Beclin-1 protein expression. (**C**) Representative Western blot images of ATG5 and β-actin. (**D**) Densitometric analysis of ATG5 protein expression. (**E**) Representative Western blot images of ATG7 and β-actin. (**F**) Densitometric analysis of ATG7 protein expression. (**G**) Representative Western blot images of p62 and β-actin. (**H**) Densitometric analysis of p62 protein expression (Data were analyzed using the Kruskal–Wallis H test followed by Bonferroni-corrected Mann–Whitney U tests for pairwise comparisons. Data are presented as box-and-whisker plots with individual data points overlaid. The box represents the IQR, the horizontal line indicates the median, and the whiskers represent the minimum and maximum values. Different letters indicate statistically significant differences between groups (*p* < 0.05), whereas groups sharing the same letter are not significantly different.

The effects of agomelatine on autophagy-related protein expression in brain tissue are presented in Figure 8. Statistical analysis demonstrated that CI/R injury significantly reduced Beclin-1, ATG5, and ATG7 protein levels, while increasing p62 protein levels compared with the sham group (*p* < 0.05). Agomelatine treatment significantly reversed these alterations, resulting in elevated Beclin-1, ATG5, and ATG7 expression and reduced p62 levels relative to the CI/R group (*p* < 0.05). The increase in Beclin-1 expression and the decrease in p62 expression were dose-dependent, with the 40 mg/kg agomelatine group exhibiting greater effects than the 20 mg/kg group (*p* < 0.05). Similarly, ATG5 protein levels were significantly higher in the 40 mg/kg agomelatine group than in the CI/R group and reached values comparable to those of the sham group (*p* > 0.05). In contrast, although agomelatine significantly increased ATG7 protein levels, no dose-dependent difference was observed between the two treatment groups (*p* > 0.05).

## 4. Discussion

### 4.1. Effects of Agomelatine on Neurological Deficits, Infarct Area, and Behavioral Outcomes Following CI/R Injury

CI/R is a pathophysiological process characterized by extensive neuronal injury resulting from the reduction and/or interruption of cerebral blood flow due to the occlusion of blood vessels supplying the brain. The ability of agomelatine to promote neuronal proliferation and neurogenesis, attenuate neuroinflammation, and improve metabolic parameters implicated in the pathogenesis of CI/R suggests that it may exert protective effects against CI/R-induced injury. The limited number of studies investigating the effects of agomelatine on CI/R injury have demonstrated that agomelatine treatment reduces neurological deficits, infarct area, and somatosensory impairment, while improving balance and motor coordination [19,28]. To our knowledge, no studies have specifically investigated the effects of agomelatine on CI/R-induced impairments in grip strength. However, agomelatine has been reported to improve balance, motor coordination, and grip strength in experimental models of Huntington’s disease, a neurodegenerative disorder [29,30], as well as under anxiogenic conditions [31]. Furthermore, several studies have demonstrated that ramelteon, another melatonin receptor agonist with pharmacological properties similar to those of agomelatine, attenuates CI/R-induced infarct formation and neurological deficits [32]. Previous studies have demonstrated that melatonin administration attenuates CI/R-induced neurological deficits, infarct area, and somatosensory impairment, as evidenced by reduced NDS and adhesive removal time, while simultaneously improving balance and motor coordination, reflected by increased rotarod latency [33]. These findings suggested that agomelatine, a melatonin receptor agonist, may also possess therapeutic potential in mitigating CI/R-induced injury. In the present study, agomelatine was administered at the onset of reperfusion, a critical phase characterized by excessive reactive oxygen species generation, inflammatory responses, and mitochondrial dysfunction. Importantly, the timing of agomelatine administration in the present study differs from that of previous studies, in which the drug was administered either before the induction of ischemia or at the second hour of reperfusion. By administering agomelatine at the onset of reperfusion, the present study more closely mimics a clinically relevant therapeutic intervention and provides additional evidence for its neuroprotective potential during the early reperfusion phase. Therefore, early administration of agomelatine may have contributed to the observed neuroprotective effects by limiting reperfusion-associated neuronal damage. Consistent with this notion, agomelatine administration reduced NDS, infarct area, and adhesive removal time in rats subjected to MCAO-induced CI/R, while significantly increasing rotarod latency and grip strength. However, it should be noted that infarct area assessment by TTC staining was performed using a relatively small number of animals (n = 3 per group), which may have limited the statistical power of this analysis. Therefore, these findings should be interpreted with caution and confirmed in future studies with larger sample sizes. These findings are in agreement with previous reports and indicate that agomelatine attenuates neurological deficits and infarct size following CI/R injury. Furthermore, agomelatine improved CI/R-induced somatosensory impairment, balance and motor coordination deficits, and reductions in grip strength. Notably, the 40 mg/kg dose produced greater improvements in adhesive removal performance and grip strength than the 20 mg/kg dose, suggesting a dose-dependent beneficial effect of agomelatine on functional recovery following CI/R injury.

### 4.2. Effects of Agomelatine on Apoptosis

Several studies have demonstrated that agomelatine inhibits apoptosis in peripheral tissues by increasing the levels of anti-apoptotic proteins, such as Bcl-2 and Bcl-XL, while reducing the expression of pro-apoptotic proteins, including Bax, cytochrome c, and caspase-3 [18]. In vitro studies demonstrating that agomelatine reduces caspase-3 and caspase-9 protein levels in hippocampal neurons, together with in vivo studies showing that agomelatine decreases Bax expression while increasing Bcl-2 levels in hippocampal tissue subjected to endoplasmic reticulum stress, further support the anti-apoptotic effects of agomelatine within the central nervous system [34,35]. Evidence indicating that melatonin mitigates CI/R-induced injury by suppressing apoptotic pathways further supports the hypothesis that agomelatine, owing to its melatonergic receptor agonism, may possess comparable anti-apoptotic and neuroprotective properties [36]. Previous studies examining the effects of agomelatine in CI/R models have demonstrated that agomelatine attenuates CI/R-induced apoptotic activation by increasing the expression of the anti-apoptotic proteins Bcl-2 and Bcl-XL and decreasing the levels of the pro-apoptotic proteins Bax and caspase-3 [19,37]. Consistent with these findings, the present study demonstrated that CI/R injury significantly decreased Bcl-2 protein expression and increased Bax levels in brain tissue. Agomelatine treatment reversed these alterations, resulting in elevated Bcl-2 expression and reduced Bax expression. Moreover, the reduction in Bax protein levels was more pronounced in the 40 mg/kg agomelatine group, suggesting that the anti-apoptotic effects of agomelatine may be dose-dependent. These findings suggest that agomelatine may attenuate apoptosis-related signaling and preserve neuronal integrity following cerebral ischemia/reperfusion injury. However, although Bcl-2 and Bax are widely used markers of apoptotic signaling, additional analyses, such as caspase activation assays or TUNEL staining, would be required to more definitively confirm the anti-apoptotic effects of agomelatine.

### 4.3. Effects of Agomelatine on Autophagy

Hypoxia, ATP depletion, and mitochondrial dysfunction, which are characteristic features of CI/R pathophysiology, represent key stimuli for the activation of autophagy [38]. Previous studies have demonstrated that autophagy activation may attenuate cellular injury during CI/R by promoting energy generation and facilitating the removal of damaged cellular components [39,40]. Yılmaz et al. reported that the beneficial effects of melatonin in CI/R injury may be mediated, at least in part, through the activation of autophagy-related pathways [33]. Previous studies have demonstrated that agomelatine promotes autophagy and exerts neuroprotective effects in U87-MG and A172 glioblastoma cells and in the hippocampal tissue of stress-exposed mice [41]. Nevertheless, the contribution of autophagy to the protective effects of agomelatine in CI/R injury remains largely unexplored, representing an important gap in the literature. Collectively, our findings indicate that agomelatine may alleviate CI/R-induced injury by restoring autophagic activity, as evidenced by increased Beclin-1, ATG5, and ATG7 levels and decreased p62 accumulation. Moreover, the 40 mg/kg dose exerted more pronounced effects on Beclin-1, ATG5, and p62 expression than the 20 mg/kg dose, suggesting that the regulation of autophagy by agomelatine may be dose-dependent. Therefore, the present study provides important preliminary evidence supporting a potential role for autophagy induction in the neuroprotective effects of agomelatine following CI/R injury. Moreover, the combined assessment of grip strength and multiple autophagy-related proteins, including Beclin-1, ATG5, ATG7, and p62, provides novel mechanistic insights into the neuroprotective actions of agomelatine.

## 5. Conclusions

The present study demonstrated that agomelatine improved neurological and behavioral outcomes following CI/R injury, as evidenced by increased rotarod performance and grip strength, reduced adhesive removal time, and decreased infarct area. Furthermore, agomelatine increased Bcl-2 expression while reducing Bax levels, suggesting an anti-apoptotic effect. Agomelatine also increased the levels of Beclin-1, ATG5, and ATG7 and decreased p62 expression, indicating the induction of autophagic activity. Taken together, these findings suggest that agomelatine exerts neuroprotective effects against CI/R-induced brain injury, potentially through the suppression of apoptosis and the promotion of autophagy. However, given the complex pathophysiology of CI/R, further studies are needed to clarify the molecular mechanisms underlying these effects. Future research investigating different doses, routes of administration, reperfusion durations, and the signaling pathways associated with apoptosis and autophagy may provide a more comprehensive understanding of the therapeutic potential of agomelatine in ischemic stroke.

## Figures and Tables

**Table 1:** Neurological Deficit Scoring according to the Bederson method.

Neurological Status	Score
Healthy neurological condition (no deficit)	0 point
Flexion in the contralateral forelimb when lifted by the tail	1 point
Flexion in the contralateral forelimb and decreased resistance to lateral push	2 points
Circling toward the contralateral side while walking	3 points
Spinning movement or seizure activity when lifted by the tail	4 points
Inability to walk spontaneously	5 points

## Data Availability

The data presented in this study are available from the corresponding author upon reasonable request.

## Abbreviations

ATG5	Autophagy Related 5
ATG7	Autophagy Related 7
Bax	Bcl-2-Associated X Protein
Bcl-2	B-Cell Lymphoma 2
BCA	Bicinchoninic Acid
BDNF	Brain-Derived Neurotrophic Factor
CCA	Common Carotid Artery
CI	Cerebral Ischemia
CI/R	Cerebral Ischemia/Reperfusion
ECA	External Carotid Artery
ECL	Enhanced Chemiluminescence
HEC	Hydroxyethyl Cellulose
HRP	Horseradish Peroxidase
ICA	Internal Carotid Artery
MCA	Middle Cerebral Artery
MCAO	Middle Cerebral Artery Occlusion
MT1	Melatonin Receptor Type 1
MT2	Melatonin Receptor Type 2
NDS	Neurological Deficit Score
PBS	Phosphate-Buffered Saline
RIPA	Radioimmunoprecipitation Assay Buffer
rpm	Revolutions Per Minute
TTC	2,3,5-Triphenyltetrazolium Chloride
TBS-T	Tris-Buffered Saline with Tween 20
5-HT2C	5-Hydroxytryptamine 2C Receptor
